# Multidimensional tumor heterogeneity and its role in therapeutic resistance

**DOI:** 10.3389/fimmu.2026.1794130

**Published:** 2026-03-25

**Authors:** Nida Mubin, Mohammed Alnukhali, Nayab Ahmad, James Joseph Driscoll, Anis Ahmad

**Affiliations:** 1 Division of Hematology Oncology, Case Western Reserve University School of Medicine, Cleveland, OH, United States; 2 Department of Radiation Oncology, University of Miami Miller School of Medicine, Sylvester Comprehensive Cancer Center, Miami, FL, United States; 3 Department of Biochemistry and Molecular Biology, University of Miami Miller School of Medicine, Sylvester Comprehensive Cancer Center, Miami, FL, United States; 4 Case Comprehensive Cancer Center, Case Western Reserve University, Cleveland, OH, United States

**Keywords:** tumor heterogeneity, therapeutic resistance, tumor microenvironment, myeloid cell plasticity, cancer stem cells, clonal evolution, spatial transcriptomics, liquid biopsy

## Abstract

Tumor heterogeneity is a fundamental driver of therapeutic resistance across solid malignancies, arising from genetic, epigenetic, phenotypic, spatial, temporal, and microenvironmental diversity. In tumors developing at mucosal barrier sites, these heterogeneous features are further shaped by the unique immunological context of mucosal tissues, where immune tolerance, chronic inflammation, and continuous antigen exposure create permissive environments for immune escape and adaptive resistance. Accumulating evidence indicates that myeloid cell plasticity, including functional diversification of granulocytes, macrophages, monocytes, and dendritic cells, represents a critical interface between tumor-intrinsic heterogeneity and mucosal immune regulation. These myeloid populations contribute to spatially organized immunosuppressive niches, altered antigen processing and presentation, and therapy-induced immune remodeling, collectively influencing responses to chemotherapy, targeted therapy, and immunotherapy. Advances in single-cell sequencing, spatial transcriptomics, multiplex imaging, and liquid biopsy technologies, coupled with artificial intelligence-enabled analytics, have enabled high-resolution mapping of heterogeneous tumor immune landscapes and revealed convergent resistance mechanisms driven by clonal selection, phenotypic plasticity, microenvironmental buffering, and myeloid-mediated immune suppression. In this review, we synthesize mechanistic and clinical evidence across major cancer types, including colorectal and lung cancers as archetypal mucosal tumors, along with broader examples from breast cancer, melanoma, and immunotherapy-treated malignancies. We highlight how heterogeneous cellular states and immune niches influence clinical outcomes. Finally, we discuss emerging translational strategies to overcome resistance, including rational combination regimens, epigenetic and metabolic targeting, adaptive therapy, myeloid reprogramming approaches, and real-time biomarker monitoring. These approaches aim to restore effective anti-tumor immunity while accounting for the unique constraints of mucosal barrier tissue.

## 1 Introduction

Over the past two decades, significant advances in cancer therapy, including molecularly targeted agents, immune checkpoint inhibitors, and precision oncology strategies, have improved initial response rates and short-term clinical outcomes across multiple malignancies (1). However, for most solid tumors, these responses remain transient, with most patients ultimately experiencing disease progression and relapse. Consequently, therapeutic resistance, rather than initial lack of efficacy, has emerged as the dominant clinical barrier to durable cancer control and long-term survival (2). A growing body of clinical, genomic, and longitudinal evidence indicates that single molecular alterations do not drive resistance. Instead, it arises from extensive tumor heterogeneity operating across genetic, epigenetic, phenotypic, metabolic, spatial, temporal, and microenvironmental dimensions (3). This heterogeneity enables parallel evolutionary trajectories under therapeutic pressure, allowing resistant subpopulations to persist, adapt, and ultimately dominate, thereby undermining the durability of chemotherapy, targeted therapy, and immunotherapy alike (4).

### 1.1 The central challenge: tumor heterogeneity as the root cause of therapeutic failure

Cancer progression and treatment failure are best understood through the lens of cellular heterogeneity rather than uniform malignant behavior. In contrast to normal tissues, where tightly regulated developmental and homeostatic programs constrain lineage identity and function, tumors comprise diverse populations of cancer cells that differ across genetic, epigenetic, transcriptional, metabolic, and phenotypic axes (5). These differences are further structured in space and time by gradients in oxygen, nutrients, immune surveillance, and therapeutic exposure within the tumor microenvironment (TME), generating a dynamic, evolving ecosystem rather than a static disease entity (6).

This multidimensional heterogeneity is not a secondary feature of cancer biology but a primary determinant of therapeutic outcome. Clinical resistance arises because anticancer therapies rarely eliminate all malignant cell states simultaneously (7). Instead, treatment imposes selective pressure that favors the survival and expansion of pre-existing resistant subclones, induces adaptive phenotypic state transitions, or enables cancer cells to exploit protective microenvironmental niches (8–10). As a result, even therapies engineered for high molecular specificity, such as kinase inhibitors or immune checkpoint blockade, frequently produce only transient disease control, followed by relapse driven by evolutionary escape. Durable remissions therefore remain the exception rather than the rule in most advanced solid malignancies (11, 12).

### 1.2 Historical perspective and the evolution of the heterogeneity paradigm

The recognition of tumors as heterogeneous systems predates modern genomics. Seminal experimental studies in the 1970s, notably by Fidler and colleagues, demonstrated that metastatic potential varied among clonal populations derived from the same primary tumor, establishing early evidence that malignancies consist of biologically distinct subpopulations (13). However, the clinical significance of this observation remained underappreciated for decades, in part because available technologies could not adequately resolve intratumoral diversity. The first wave of large-scale cancer genome sequencing revealed extensive mutational variability within individual tumors, challenging the prevailing assumption of molecular uniformity (14). Yet these early efforts were constrained by bulk sequencing approaches that averaged signals across millions of cells, obscuring rare but clinically consequential subclones (15, 16) Consequently, both cytotoxic chemotherapy and early targeted therapies were developed under an essentially reductionist framework, implicitly if single molecular lesions identified from a single biopsy could define tumor behavior and guide durable treatment decisions (17, 18).

This paradigm has shifted decisively with the advent of high-resolution and longitudinal profiling technologies. Single-cell RNA sequencing has enabled the routine analysis of tens to hundreds of thousands of individual tumors and microenvironmental cells, uncovering coexisting transcriptional programs and lineage states within the same lesion (19–21). Spatial transcriptomics and multiplexed imaging preserve tissue architecture while resolving gene and protein expression, revealing how tumor geography shapes cellular behavior and therapeutic vulnerability (22, 23). Liquid biopsy approaches that quantify circulating tumor DNA (ctDNA) now allow real-time tracking of clonal dynamics and resistance evolution during therapy. Importantly, artificial intelligence (AI)–based analytical frameworks integrate these high-dimensional datasets to extract patterns and trajectories that are not discernible through conventional analysis (21, 24). Collectively, these advances have reframed cancer as an evolving, adaptive system governed by ecological and evolutionary principles. They underscore the need for combination therapies, longitudinal biomarker assessment, and adaptive treatment strategies to counteract heterogeneity-driven resistance.

### 1.3 Scope and objectives

Here, we systematically examine tumor heterogeneity as the central biological mechanism underlying therapeutic resistance. We categorize the origins and manifestations of heterogeneity across genetic, epigenetic, phenotypic, spatial, temporal, and microenvironmental dimensions and delineate the mechanistic pathways through which each contributes to drug resistance. Building on these insights, we then contextualize these principles using clinically relevant examples from major cancer types illustrate how heterogeneity-driven resistance emerges in patients treated with chemotherapy, targeted therapies, and immunotherapy. Further, we then evaluate emerging strategies to anticipate and constrain tumor evolution, including adaptive therapy, epigenetic and plasticity targeting, microenvironmental modulation, and rational combination and sequencing regimens. Finally, we discuss future directions in precision oncology, integrating multimodal profiling, computational modeling, and artificial intelligence to enable evolution-aware therapeutic decision-making. Notably, the various dimensions of tumor heterogeneity differ in their accessibility and relevance to therapeutic intervention. Translational advances to date have been most pronounced for a defined subset of actionable heterogeneity features, including (i) truncal oncogenic drivers and convergent resistance mutations, (ii) tumor immune contexture, (particularly inflamed versus immune-excluded states), (iii) myeloid-mediated immunosuppressive niches within the tumor microenvironment, as a modifiable resistance axis, (iv) epigenetic and phenotypic plasticity that supports reversible drug-tolerant states, and (v) spatially organized hypoxic and stromal niches that influence drug delivery and immune accessibility. Accordingly, this review prioritizes these clinically actionable forms of heterogeneity and systematically connects them to established and emerging therapeutic strategies.

### 1.4 Origins and manifestations of tumor heterogeneity

**Figure 1:**
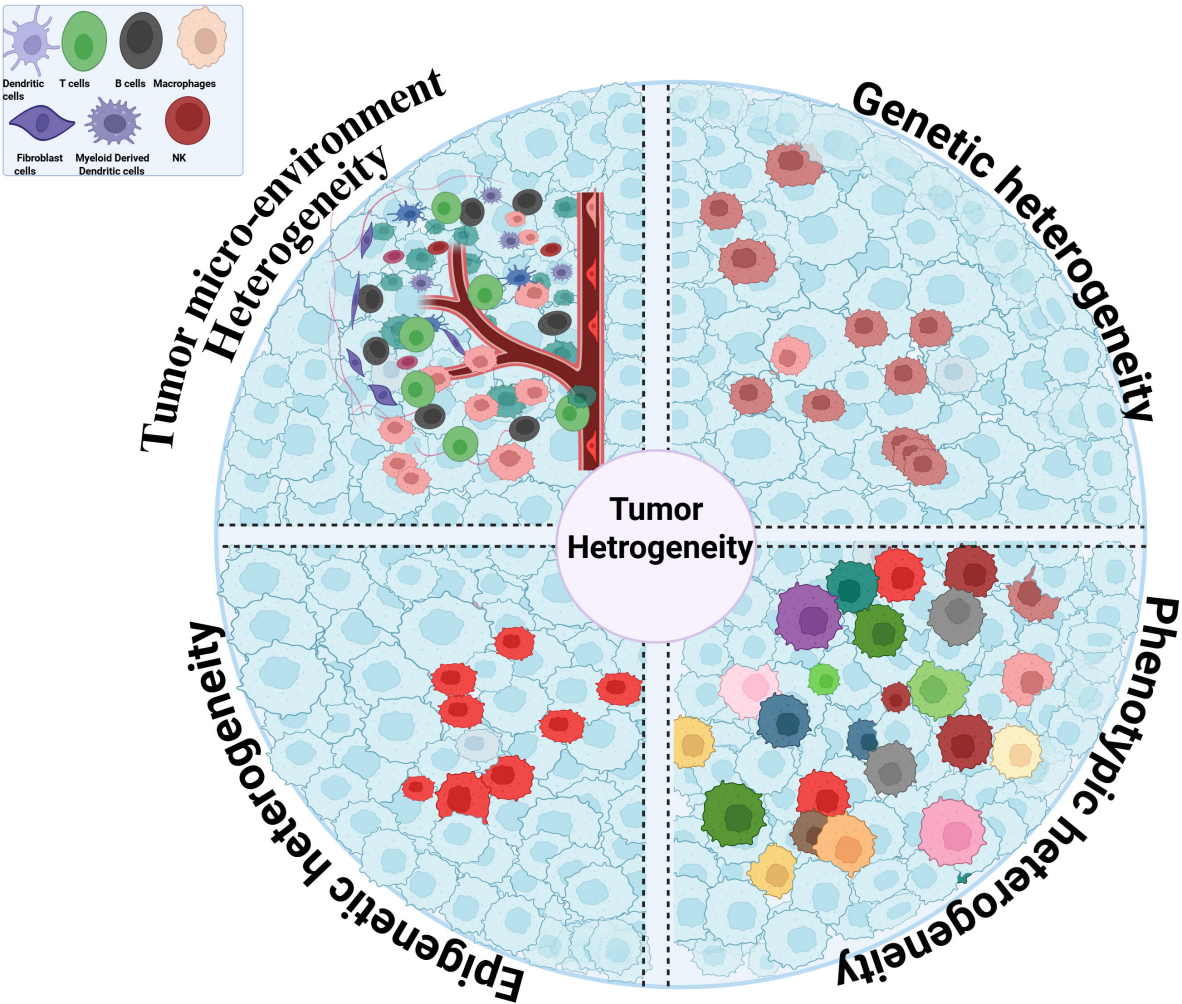
Multidimensional architecture of tumor heterogeneity as an integrated biological system. Schematic representation of the major biological dimensions of tumor heterogeneity, including genetic, epigenetic, phenotypic, and tumor microenvironmental heterogeneity. These layers coexist within individual tumors and interact dynamically to generate diverse cellular states, clonal architectures, and adaptive behaviors. The convergence of these heterogeneous processes underlies differential therapeutic responses, promotes resistance evolution, and drives disease progression. TME, tumor microenvironment.

Tumor heterogeneity arises from the interplay of genetic diversification, epigenetic regulation, transcriptional plasticity, and microenvironmental conditioning. These processes operate across both spatial and temporal scales (25). These interconnected dimensions coexist within individual tumors and across patients, encompassing genetic, epigenetic, phenotypic, and microenvironmental layers, and are summarized in **Figure 1**. Together, they generate malignant cell populations with distinct biological properties, evolutionary fitness, and therapeutic sensitivities (26). Importantly, heterogeneity is embedded not only between patients but also within individual tumors. Within tumors diverse cancer cells dynamically interact with stromal and immune components, shaping treatment response and clinical outcome (27, 28).

## 2 Intertumoral and intratumoral heterogeneity: distinct but interconnected dimensions

Intertumoral heterogeneity refers to phenotypic and molecular variation between tumors arising in different patients with the same histopathological diagnosis. Even within narrowly defined disease categories, such as hormone receptor–positive breast cancer, large-scale genomic and transcriptomic profiling efforts have revealed extensive diversity (29, 30). Studies from large consortia, using CIBERSORT analysis of TCGA and METABRIC, database have identify prognostically distinct subgroups of triple-negative breast cancer with improved clinical outcomes. These findings demonstrate that tumors sharing identical receptor status often harbor distinct constellations of driver mutations, copy number alterations, and transcriptional programs, resulting in variable therapeutic vulnerabilities (31). This interpatient variability has motivated precision oncology initiatives, including tumor boards and basket trial designs, which assign therapies based on actionable alterations rather than tissue of origin (32, 33). However, intertumoral heterogeneity represents only one axis of complexity. Intratumoral heterogeneity (ITH), the coexistence of genetically and phenotypically distinct cancer cell populations within a single tumor, poses a more formidable challenge to durable therapeutic control (34). ITH manifests along both spatial and temporal dimensions. Spatially, different regions of the same tumor can harbor divergent genomic alterations and expression programs; temporally, tumor composition evolves over the course of disease progression and treatment (34, 35). Seminal multi-region sequencing studies, most notably in Glioblastoma and renal cell carcinoma, demonstrated that a substantial fraction of somatic mutations is subclonal and region-specific rather than universally shared (36–38). This finding revealed that a single biopsy often fails to capture the full genomic landscape of a tumor. Across solid tumors, 30–60% of detectable mutations are heterogeneously distributed within individual tumors (39–41).

These findings fundamentally challenge the assumption that a single biopsy captures tumor-defining molecular behavior and highlights the limitations of static biomarker driven therapies. Importantly, the extent of ITH varies across malignancies, with glioblastoma, non-small cell lung cancer, and renal cell carcinoma exhibiting particularly high spatial diversity, whereas some pediatric tumors display comparatively homogeneous genomic landscapes (38, 42).

### 2.1 Spatial heterogeneity: tumor geography as a determinant of cellular state

Spatial heterogeneity reflects the structured organization of tumor cell states across distinct microenvironmental niches. Gradients in oxygen availability, nutrient supply, metabolic byproducts, immune surveillance, and drug penetration generate localized selective pressures that favor specific phenotypes (43, 44).Cells proximal to functional vasculature typically experience normoxic, nutrient-rich conditions that support high proliferative capacity and relative sensitivity to cytotoxic and antimetabolic therapies (45–47).Spatial transcriptomics in breast cancer TMEs reveal that regions closer to functional vasculature and the tumor periphery show increased proliferative and oxidative metabolic programs, correlating with distinct drug-sensitivity gradients relative to hypoxic tumor cores (46). These recent studies provide direct evidence that proximity to functional blood vessels creates microenvironmental niches with greater oxygen and nutrient availability, supporting highly proliferative tumor cells and influencing therapeutic responsiveness. In contrast, cells residing in poorly perfused regions are exposed to hypoxia, acidosis, and metabolic stress, conditions that induce adaptive stress-response programs and promote resistance-associated phenotypes (33, 48–50). Advances in spatial transcriptomics and multiplexed imaging have enabled direct visualization of these patterns. In pancreatic ductal adenocarcinoma, for example, spatial profiling has revealed compartmentalized transcriptional programs: proliferative states are enriched in vascularized regions, hypoxia- and glycolysis-associated programs dominate the tumor core, and mesenchymal or invasive states are concentrated at the tumor-stroma interface. Immune infiltration follows similarly structured patterns, with sharply demarcated regions of immune-rich (“inflamed”) and immune-excluded (“cold”) tumor architecture often coexisting within the same lesion (51, 52).

This spatial organization is not stochastic but reflects ecological principles. Cancer cells adapt to local resource constraints, forming stable niches analogous to those observed in natural ecosystems. Phenotypes optimized for hypoxic survival are typically maladapted to normoxic environments, reinforcing spatial segregation. Notably, transitional zones between niches where environmental gradients are steepest often exhibit heightened cellular plasticity and evolutionary potential (53, 54). Clinically, this spatial diversity can manifest as heterogeneous estrogen receptor (ER) expression and immune infiltration within a single breast tumor, leading to region-specific sensitivity and resistance to endocrine or immunotherapies.

### 2.2 Temporal heterogeneity: evolution under therapeutic pressure

Temporal heterogeneity reflects the dynamic evolution of tumors over time, particularly under therapeutic pressure. Tumors undergo continuous clonal remodeling driven by clonal expansion, extinction, mutation acquisition, and phenotypic state transitions. Resistant subclones may preexist at very low frequencies, often below the detection limits of conventional bulk sequencing but can rapidly expand when therapy imposes selective pressure (55, 56).

Longitudinal monitoring using liquid biopsy has provided compelling evidence for this evolutionary process in patients. In colorectal cancer treated with anti-EGFR antibodies, activating RAS mutations can be detected in circulating tumor DNA months before radiographic progression, indicating that molecular resistance precedes clinical failure (57–59). Similarly, in EGFR-mutant non-small cell lung cancer, resistance mutations such as T790M emerge in patient specific temporal patterns, reflecting differences in evolutionary trajectories and selective pressures (60). Importantly, temporal heterogeneity is context dependent and, in some cases, reversible. Upon withdrawal of therapy, resistant clones may decline in frequency as drug-sensitive populations regain a competitive advantage, a phenomenon termed competitive release. This principle underlies adaptive therapy strategies, which aim to control tumor burden by modulating selective pressure rather than pursuing maximal cytotoxicity (61). Conversely, sequential therapeutic regimens can drive stepwise evolutionary adaptation, exemplified by the progressive emergence of resistance mechanisms across successive generations of tyrosine kinase inhibitors in EGFR-driven lung cancer (62–65).

### 2.3 Genetic determinants of tumor heterogeneity

#### 2.3.1 Somatic mutations and clonal architecture

Genetic heterogeneity originates from the elevated mutational burden characteristic of malignant cells, driven by defects in genome maintenance pathways, replication stress, oncogene-induced DNA damage, and exposure to endogenous and exogenous mutagens. Impairment of mismatch repair, homologous recombination deficiencies associated with BRCA1/2 loss, polymerase proofreading defects (e.g., POLE mutations), and carcinogenic insults such as tobacco smoke, ultraviolet radiation, and prior chemotherapy collectively accelerate mutation accumulation and diversify tumor cell populations (66–68). These mutations are organized into a hierarchical clonal architecture reflecting temporal sequence of tumor evolution. Early acquired truncal (clonal) alterations are shared by all cancer cells and include canonical driver events affecting tumor suppressors (e.g., *TP53*, *APC*, *RB1*) and oncogenes (e.g., *KRAS*, *BRAF*, *PIK3CA*, *MYC* amplification) that establish and sustain the malignant state (69) (70) (71). In contrast, branch (sub clonal) mutations arise later and are restricted to subsets of cells, often conferring context-dependent fitness advantages. Among these are therapy-induced resistance mutations, such as *EGFR* T790M or *ABL* T315I, which may exist at low frequencies prior to treatment and expand under selective pressure (72–75). Superimposed on these functional alterations are large numbers of passenger mutations, which are largely neutral but serve as molecular records of evolutionary history (76, 77). Phylogenetic analyses have revealed that most solid tumors evolve through branched trajectories with coexisting subclones (78). Greater clonal branching correlates with poor outcomes, although genetic complexity alone does not uniformly predict therapeutic response, highlighting the need to integrate genetic, phenotypic, and microenvironmental context (26, 79, 80).

#### 2.3.2 Chromosomal instability and copy number heterogeneity

Beyond point mutations, chromosomal instability (CIN) constitutes a major engine of genetic heterogeneity through recurrent whole-chromosome gains and losses, segmental amplifications, deletions, and structural rearrangements. Single-cell whole-genome sequencing studies have demonstrated that even clonally related tumor cells can exhibit striking karyotypic diversity, with divergent chromosomal complements arising from a single mitotic division, generates a continuous supply of genetic variants for selection can act (81, 82). CIN exerts context-dependent effects on tumor fitness. Moderate instability promotes adaptation by enabling oncogene amplifications (e.g., ERBB2, CCND1, MYC) or loss of tumor suppressor loci, facilitating therapeutic escape, whereas excessive instability compromises viability. Evidence supports an optimal CIN window that maximizes evolvability while avoiding genomic collapse (83, 84). Clinically, elevated CIN correlates with poor prognosis and resistance. Therapeutically, CIN represents a paradoxical target, creating vulnerabilities to agents disrupting mitotic checkpoints, centrosome clustering, or DNA damage tolerance pathways (85, 86).

### 2.4 Epigenetic heterogeneity and phenotypic plasticity

While genetic alterations provide heritable diversity, epigenetic mechanisms enable rapid and reversible modulation of gene expression without changes to DNA sequence. This regulatory flexibility underlies normal developmental processes and is co-opted by cancer cells to adapt dynamically to environmental stress and therapeutic pressure.

#### 2.4.1 DNA methylation landscapes

DNA methylation patterns exhibit pronounced intratumoral variability. Single-cell bisulfite sequencing has revealed substantial cell-to-cell heterogeneity across thousands of CpG sites, particularly within regulatory regions governing differentiation, invasion, and drug response. Aberrant CpG island hypermethylation silences tumor suppressors and DNA repair genes (e.g., *MLH1*, *BRCA1*, *CDKN2A*), giving rise to methylator phenotypes with distinct clinical behaviors (87, 88). In parallel, global DNA hypomethylation destabilizes chromatin architecture and promotes chromosomal instability further increasing genetic diversity (89). Importantly, focal methylation variability generates transcriptionally distinct subpopulations within the same tumor., In addition to DNA methylation, non-coding RNAs, including microRNAs, long non-coding RNAs, and circular RNAs, collectively add an additional layer of post-transcriptional regulation, that contributes to heterogeneous therapeutic responses (90–93).

#### 2.4.2 Histone modifications and chromatin accessibility

Histone post-translational modifications dynamically regulate chromatin accessibility and transcriptional output. Single-cell chromatin accessibility profiling demonstrates marked heterogeneity among cancer cells with identical genotypes, revealing stable regulatory regions alongside highly plastic domains enriched near genes controlling stress response, lineage commitment, and differentiation, enabling rapid phenotypic switching (89, 94–98).

Within tumors, stable regulatory regions coexist with highly plastic chromatin domains. These plastic regions are frequently enriched near genes involved in stress responses, lineage commitment, and differentiation, allowing cancer cells to rapidly alter transcriptional programs in response to environmental or therapeutic cues (97, 98). Such epigenetic flexibility supports phenotypic switching without requiring new genetic alterations. Polycomb repressive complexes, particularly PRC2, exhibit heterogeneous activity, while EZH2 dysregulation and mutations in chromatin remodelers such as SWI/SNF further diversify chromatin states, transcriptional programs, and therapeutic sensitivities (99). Additional diversity arises from mutations or functional disruption of chromatin-remodeling complexes, including SWI/SNF family members. These alterations reshape nucleosome positioning and enhancer accessibility, further diversifying transcriptional states and therapeutic sensitivities within tumors. Collectively, heterogeneous histone modifications and chromatin accessibility landscapes provide a mechanistic basis for epigenetically driven resistance that operates alongside genetic heterogeneity and phenotypic plasticity.

#### 2.4.3 Phenotypic plasticity and cellular state transitions

The integration of genetic and epigenetic diversity gives rise to phenotypic plasticity, defined as the capacity of cancer cells to transition between distinct, interconvertible cellular states (100, 101). Rather than existing as fixed identities, tumor cells occupy a dynamic spectrum of transcriptional and functional programs. Single-cell transcriptomic analyses show tumors comprise heterogeneous populations spanning proliferative, invasive, stem-like, senescent, and drug-tolerant states rather than fixed identities (81). These states differ markedly in therapeutic sensitivity and biological function. Proliferative states are typically therapy-sensitive, whereas mesenchymal or invasive states confer migratory capacity and enhanced resistance. Stem-like states enable persistence through self-renewal, quiescence, and drug efflux, while drug-tolerant persister cells survive treatment and drive relapse. In contrast, drug-tolerant persister cells represent a transient, non-genetic survival state that enables short-term tolerance to therapy. While persister cells lack stable stem cell properties, they can act as a reservoir from which resistant or stem-like populations re-emerge following treatment.

Cancer stem cells and drug-tolerant persisters represent overlapping but distinct non-genetic resistance states: stemness is defined by durable self-renewal and tumor-propagating capacity, whereas persisters are defined by reversible survival under therapy. Stem-like states enable persistence through self-renewal, quiescence, and drug efflux, while drug-tolerant persister cells enable short-term survival under therapy and can contribute to relapse. Lineage-tracing demonstrates dynamic interconversion driven by TME cues, fundamentally limiting single-state targeting strategies (102). Collectively, these lineage tracing and longitudinal profiling reveal that these cellular states are dynamically interconvertible and strongly influenced by microenvironmental cues, including hypoxia, inflammatory signaling, and therapeutic stress (102). This bidirectional plasticity fundamentally limits strategies that target single cellular states in isolation and underscores phenotypic plasticity as a central non-genetic mechanism of therapeutic resistance.

### 2.5 Tumor microenvironment driven heterogeneity

The tumor microenvironment (TME) is a central driver of intratumoral heterogeneity and comprises diverse immune cells, endothelial, fibroblasts, pericytes, extracellular matrix components, and soluble factors, that vary in composition and function across tumors and within individual lesionsic (103, 104). Through continuous reciprocal interactions with cancer cells, the TME forms a dynamic ecosystem that shapes cellular state, evolutionary fitness, and therapeutic response.

Microenvironmental heterogeneity arises from both the intrinsic diversity of stromal and immune compartments and spatial gradients in oxygen, nutrients, metabolites, and cytokines. These factors impose selective pressures that favor distinct cancer cell phenotypes and enable the coexistence of therapy-sensitive and therapy-resistant populations within the same tumor. As a result, the TME not only reflects tumor heterogeneity but actively reinforces and amplifies it during disease progression and treatment (103, 104).

#### 2.5.1 Cancer-associated fibroblasts and stromal diversity

Cancer-associated fibroblasts (CAFs) are phenotypically and functionally heterogeneous in a cancer-type–specific manner. Single-cell profiling has identified distinct CAF subsets, including myofibroblastic CAFs enriched near tumor nests that deposit dense extracellular matrix and restrict drug penetration, inflammatory CAFs that secrete cytokines activating pro-survival signaling pathways, and antigen-presenting CAFs that modulate local immune responses. Spatial compartmentalization of these subsets has direct implications for therapeutic efficacy, as stromal architecture can simultaneously shield tumor cells and promote adaptive resistance (105, 106).

Hypoxia, metabolism, and vascular niches. Inadequate vascularization generates gradients in oxygen and nutrient availability that impose strong selective pressures. Hypoxic niches activate HIF-dependent transcriptional programs that promote angiogenesis, metabolic reprogramming, invasion, and resistance to radiotherapy and chemotherapy. Vascular niches support stem-like cancer cells through endothelial-derived paracrine signals, whereas poorly perfused regions harbor dormant populations therapy resistant (107, 108).

## 3 Mechanisms of drug resistance driven by tumor heterogeneity

Tumor heterogeneity enables therapeutic resistance through interconnected genetic, epigenetic, phenotypic, and microenvironmental mechanisms that coexist within tumors and interact, as summarized in **Table 1** and **Figure 1**, to generate drug-tolerant subpopulations that enable early resistance, underscoring the need for molecularly resolved, evolution-aware treatment strategies.

**Table 1:** Dimensions of tumor heterogeneity and their clinical consequences.

Dimension of Tumor Heterogeneity	Defining Biological Features	Representative Examples / Drivers	Primary Clinical Consequences	Therapeutic Implications	Ref.
Genetic heterogeneity	Coexistence of multiple genetic subclones within a tumor	Copy number alterations, Sub clonal driver mutations;	Variable drug sensitivity; emergence of resistant clones	Limits of single-biopsy profiling; need for combination and longitudinal strategies	(5, 80)
Epigenetic heterogeneity	Reversible chromatin and DNA methylation states	Drug-tolerant persister states; histone modifications	Transient resistance without genetic change	Rationale for epigenetic priming and combination therapies	(109–111)
Phenotypic / transcriptional heterogeneity	Diverse cell states and lineage programs within tumors	EMT programs; stem-like or quiescent cells	Non-genetic therapy escape; phenotypic plasticity	Adaptive and state-targeting therapeutic approaches	(112)
Temporal heterogeneity	Dynamic changes in tumor composition over time	Therapy-induced clonal evolution; adaptive reprogramming	Progressive loss of treatment efficacy	Need for longitudinal monitoring (e.g., liquid biopsy), adaptive therapy strategies, and sequential treatment approaches	(113)
Microenvironmental heterogeneity	Variability in immune, stromal, and metabolic contexts	Myeloid-dominated niches; CAF-rich regions	Immune suppression; microenvironment-mediated protection	TME-targeted and combination immunotherapies	(113, 114)

EMT, epithelial–mesenchymal transition; CAF, cancer-associated fibroblast; TME, tumor microenvironment; DNA, deoxyribonucleic acid.

### 3.1 Pre-existing resistant clones: selection of rare but fit subpopulations

One of the most direct consequences of intratumoral genetic heterogeneity is the presence of rare, pre-existing resistant subclones prior to therapeutic exposure (115, 116). These populations often exist below the detection threshold of conventional bulk sequencing but can be identified using ultra-sensitive methods such as digital PCR, BEAMing, and ultra-deep next-generation sequencing (117). Although minor at baseline, such clones gain a decisive selective advantage after treatment initiation. In EGFR-mutant non–small cell lung cancer, the gatekeeper mutation EGFR T790M is detectable in treatment-naïve tumors at allele frequencies as low as 0.01–1% using extremely sensitive assays and rapidly expands under first-generation EGFR tyrosine kinase inhibitors to drive disease progression. Similarly, in metastatic colorectal cancer, BEAMing digital PCR of pretreatment plasma identifies low-frequency KRAS and NRAS mutations undetectable by standard tissue genotyping that accurately predict early failure of anti-EGFR monoclonal antibodies despite initial RAS wild-type classification. Pre-existing resistance also occurs beyond solid tumors; in chronic myeloid leukemia, low-frequency BCR–ABL T315I mutations at diagnosis are strongly associated with imatinib resistance. In BRAF V600E–mutant melanoma, deep and single-cell sequencing reveals multiple resistant subpopulations before therapy that emerge in parallel under BRAF/MEK inhibition, explaining the frequent failure of sequential monotherapies. The likelihood of pre-existing resistant clones is shaped by tumor size, baseline mutational burden, fitness costs, and microenvironmental constraints, making therapeutic failure an expected evolutionary outcome under strong selective pressure.

### 3.2 Clonal evolution and Darwinian selection under therapeutic pressure

Beyond the selection of rare pre-existing resistant clones, therapeutic intervention itself functions as a powerful evolutionary force that reshapes tumor architecture through Darwinian selection. Anticancer therapies impose non-physiological constraints on tumor cell populations, favoring the survival and expansion of clones with fitness advantages under drug exposure. Longitudinal and multi-region genomic profiling across cancer types provides direct clinical evidence that treatment actively redirects tumor evolutionary trajectories rather than merely revealing latent resistance.

Large prospective studies illustrate how therapy-driven evolution unfolds in patients. The TRACERx program demonstrated that targeted therapies in non–small cell lung cancer drive stepwise selection of resistant EGFR subclones over time, with distinct evolutionary paths emerging in spatially separated tumor regions. Parallel analyses in renal cell carcinoma revealed convergent evolution toward sarcomatoid differentiation despite disparate initiating mutations (60, 118, 119). In chronic myeloid leukemia, sequential tyrosine kinase inhibitor exposure selects for ordered acquisition of BCR–ABL kinase domain mutations (120). Comparable evolutionary dynamics occur across solid tumors, including RAS–MAPK pathway alterations in colorectal cancer, branching resistance in BRAF-mutant melanoma, BRCA1/2 reversion mutations under PARP inhibition in ovarian cancer, and lineage plasticity leading to neuroendocrine differentiation in prostate cancer (121, 122). These observations demonstrate that resistance emerges through branching, convergent, and phenotypic evolutionary processes. Clonal evolution under therapy intersects with adaptive non-genetic mechanisms, providing a transition to adaptive resistance mechanisms discussed below.

### 3.3 Microenvironmental protection as an extrinsic driver of drug resistance

In addition to cell-intrinsic genetic and epigenetic mechanisms, the tumor microenvironment provides extrinsic protection that enables malignant cells to withstand therapeutic pressure through reciprocal interactions with stromal, vascular, metabolic, and immune components. These microenvironment-mediated mechanisms reshape drug exposure, signaling dependencies, and survival thresholds, operating in parallel with clonal evolution and adaptive plasticity to reinforce resistance across spatial and temporal scales. Microenvironment-mediated resistance operates in parallel with clonal evolution and phenotypic plasticity. By creating protective niches, the TME buffers tumor cells from cytotoxic stress, allowing genetically diverse and phenotypically adaptable populations to persist during treatment. These protected reservoirs subsequently serve as sources of relapse once selective pressure is reduced or therapy is withdrawn (123).

#### 3.3.1 Stromal shielding and paracrine resistance signaling

Cancer-associated fibroblasts (CAFs) represent a dominant stromal source of therapeutic resistance by simultaneously restricting drug delivery and activating bypass survival pathways (124). CAF-driven extracellular matrix remodeling including excessive deposition and crosslinking of collagen and fibronectin creates dense, stiff tumor architecture that physically limits intratumoral drug penetration, as demonstrated in pancreatic ductal adenocarcinoma and breast cancer This stromal barrier results in heterogeneous drug exposure, enabling protected tumor regions to survive otherwise effective systemic therapy (125, 126). Beyond physical shielding, CAFs actively promote resistance through paracrine signaling (127). Hepatocyte growth factor secreted by CAFs activates MET signaling in cancer cells, restoring downstream MAPK and PI3K–AKT pathways and conferring resistance to BRAF inhibitors in melanoma and EGFR inhibitors in lung cancer (128, 129). CAF-derived inflammatory cytokines such as IL-6 and IL-8 further reinforce survival and stem-like programs through STAT3 and NF-κB activation in colorectal and ovarian cancers. These interactions establish stromal niches that sustain tumor viability even in the presence of potent targeted agents, directly linking microenvironmental signaling to the persistence of resistant subpopulations discussed earlier and play dual roles from senescence sentinels to death regulators as a new dimensions in therapy (130–132).

#### 3.3.2 Hypoxia, acidity, and nutrient gradients as resistance sanctuaries

Aberrant tumor vasculature generates steep gradients of oxygen, pH, and nutrients that profoundly influence therapeutic responsiveness. In solid tumors such as glioblastoma, pancreatic ductal adenocarcinoma, and head and neck squamous cell carcinoma, hypoxia stabilizes HIF-1α and HIF-2α, inducing transcriptional programs that promote angiogenesis, metabolic adaptation, survival, and invasion while diminishing sensitivity to radiotherapy and cytotoxic agents. Hypoxic tumor cells frequently enter quiescent or slow-cycling states and downregulate homologous recombination and mismatch repair pathways, rendering them intrinsically resistant to DNA-damaging therapies and antimitotic drugs (108, 133, 134). Concurrent extracellular acidosis resulting from anaerobic glycolysis and impaired perfusion reduces uptake of weakly basic chemotherapeutics through pH-dependent ion trapping, further limiting drug efficacy (135, 136).

These hypoxic and acidic regions function as spatial refuges in which genetically and phenotypically diverse tumor cells persist, reinforcing both pre-existing and adaptive resistance mechanisms and setting the stage for relapse once selective pressure is relieved.

#### 3.3.3 Drug efflux and metabolic detoxification programs

Intratumoral heterogeneity in drug handling further constrains durable therapeutic response. ATP-binding cassette transporters including ABCB1 (MDR1), ABCG2 (BCRP), and ABCC family members actively export chemotherapeutic and targeted agents, reducing intracellular drug concentrations below cytotoxic thresholds (137). Clinically, ABCB1 expression underlies resistance to taxanes and anthracyclines in breast and ovarian cancers, while ABCG2 marks stem-like populations that tolerate topoisomerase inhibitors and tyrosine kinase inhibitors in leukemia and lung cancer. In parallel, heterogeneity in drug-metabolizing enzymes reshapes treatment efficacy (138–141). Cytochrome P450 enzymes alter drug activation and clearance, glutathione S-transferases mediate platinum detoxification, and aldehyde dehydrogenases frequently enriched in stem-like populations confer broad resistance and poor prognosis in breast and prostate cancers. These efflux and detoxification programs generate uneven intracellular drug exposure across tumor cell populations, reinforcing survival of protected subsets that interface directly with stemness and plasticity-driven relapse mechanism (141, 142).

#### 3.3.4 Cancer stemness, plasticity, and therapy-induced relapse

Cancer stem cells (CSCs) and plastic stem-like states represent a convergence point for genetic, epigenetic, metabolic, and microenvironmental resistance mechanisms. CSCs are functionally defined by their capacity for long-term self-renewal, tumor propagation, and lineage reconstitution, and they possess intrinsic resistance traits, including efficient DNA damage repair, high drug efflux capacity, quiescence, and resistance to apoptosis. Importantly, stemness is not a fixed cellular identity, therapeutic stress and microenvironmental cues can induce non-stem tumor cells to transiently acquire stem-like properties through phenotypic plasticity. This dedifferentiate process is driven by the activation of developmental transcriptional programs governed by SOX2, OCT4, NANOG, and signaling pathways such as WNT, NOTCH, and Hedgehog.

In parallel, drug-tolerant persister cells represent a distinct but related non-genetic resistance state. Persisters survive acute therapeutic exposure through reversible transcriptional and metabolic adaptations rather than stable self-renewal capacity. While persister cells are typically transient, they can provide a permissive reservoir for the subsequent emergence of genetically resistant clones or durable stem-like populations once selective pressure persists. Microenvironmental factors strongly reinforce both CSC maintenance and persister survival. Hypoxia, inflammatory cytokines, extracellular matrix remodeling, and immune-suppressive niches promote stemness programs and stabilize drug-tolerant states, enabling persistence despite effective target inhibition. These protective niches facilitate survival during therapy and create conditions that favor relapse upon treatment cessation.

Collectively, CSCs, plastic stem-like states, and drug-tolerant persisters operate on overlapping temporal and functional scales to undermine durable therapeutic control. Their dynamic interconversion and microenvironmental dependence explain why eradication of dominant tumor clones alone is insufficient and highlight the need for therapeutic strategies that simultaneously constrain plasticity, disrupt supportive niches, and prevent the re-emergence of resistant cellular states.

### 3.4 Immune evasion

Immune-mediated selection imposes additional pressure that shapes both primary and acquired resistance to immunotherapy. Primary resistance often arises from low tumor immunogenicity, defective antigen presentation machinery (APM) due to loss of b2 microglobulin, components of the MHC class I complex, and (immuno)-proteasome catalytic subunits and regulators (143). Immune exclusion is elicited by dense stroma as a physical barrier and chemokine-mediated events that preclude T-cell recruitment into the tumor bed. Spatial profiling in melanoma, NSCLC, pancreatic, and colorectal cancers have revealed marked heterogeneity in immune infiltration and checkpoint ligand expression within individual tumors that may impact or correlate with clinical response to immunotherapy (144–146). Acquired resistance emerges through immune editing, loss of dominant NeoAgs, disruption of IFN signaling pathways, and progressive T-cell exhaustion accompanied by compensatory upregulation of alternative inhibitory receptors such as TIM-3, LAG-3, and TIGIT. These adaptive immune escape mechanisms do not occur uniformly but evolve alongside tumor cell heterogeneity, further restricting durable immune control and reinforcing resistance ecosystems established by stromal and metabolic protection (147, 148).

## 4 Clinical Implications of tumor heterogeneity

Evidence for multiple resistance mechanisms underscores that therapeutic failure is not driven by a single dominant pathway, but rather emerges from the interplay of genetic diversification, non-genetic plasticity, microenvironmental protection, and immune evasion operating across spatial and temporal scales (8, 11, 149). Pre-existing resistant clones, adaptive signaling reprogramming, phenotypic state transitions, and therapy-induced evolutionary selection collectively enable tumors to evade otherwise effective treatments (150). Importantly, these processes often occur in parallel within the same patient, producing polyclonal and lesion-specific resistance patterns that limit the durability of sequential monotherapies. This mechanistic framework establishes that resistance is an expected evolutionary outcome of treatment pressure acting on heterogeneous tumor ecosystems, rather than an exceptional event (151). Spatial and temporal heterogeneity undermines the accuracy of diagnostic sampling, limits the predictive value of established biomarkers, and constrains the durability of therapeutic responses, as conventional single-time-point, single-site assessments frequently fail to capture the full evolutionary landscape of disease (152, 153).

### 4.1 Diagnostic and prognostic challenges

Building on the resistance mechanisms detailed in Section 3, this section focuses on how tumor heterogeneity constrains the effectiveness of chemotherapy, targeted therapy, and immunotherapy in clinical practice.

#### 4.1.1 Biopsy limitations and spatial sampling bias

A central diagnostic challenge arises from the inherent sampling bias of tissue biopsies, which typically interrogate less than 1% of the total tumor burden. Temporal and evolutionary heterogeneity, summarized in **Table 1**, underscore the need for longitudinal and multi-site monitoring strategies. Given extensive spatial heterogeneity, single-site biopsies frequently fail to capture low frequency resistant subclones, leading to underestimation of actionable alterations and misclassification of molecular profiles. Large multi-region sequencing studies, including TRACERx Lung and TRACERx Renal, have demonstrated pronounced regional divergence in driver mutations, with therapeutically relevant alterations present in some tumor regions but absent in others (154, 155). These limitations are amplified in metastatic disease, where substantial genetic divergence between primary tumors and individual metastatic lesions has been documented across lungs, breast, Squamous Cell Carcinoma (SCC), melanoma, and prostate cancers. As a result, clinical decisions based on limited tissue sampling may not reflect the full evolutionary landscape driving therapeutic response (156).

#### 4.1.2 ctDNA versus tissue profiling: complementary views of heterogeneity

Liquid biopsy–based analysis of circulating tumor DNA (ctDNA) provides a non-invasive means to integrate genomic signals from multiple disease sites simultaneously (157). ctDNA profiling has proven particularly valuable for detecting emergent resistance mutations, monitoring minimal residual disease, and tracking clonal dynamics over time (158, 159).

**Figure 2:**
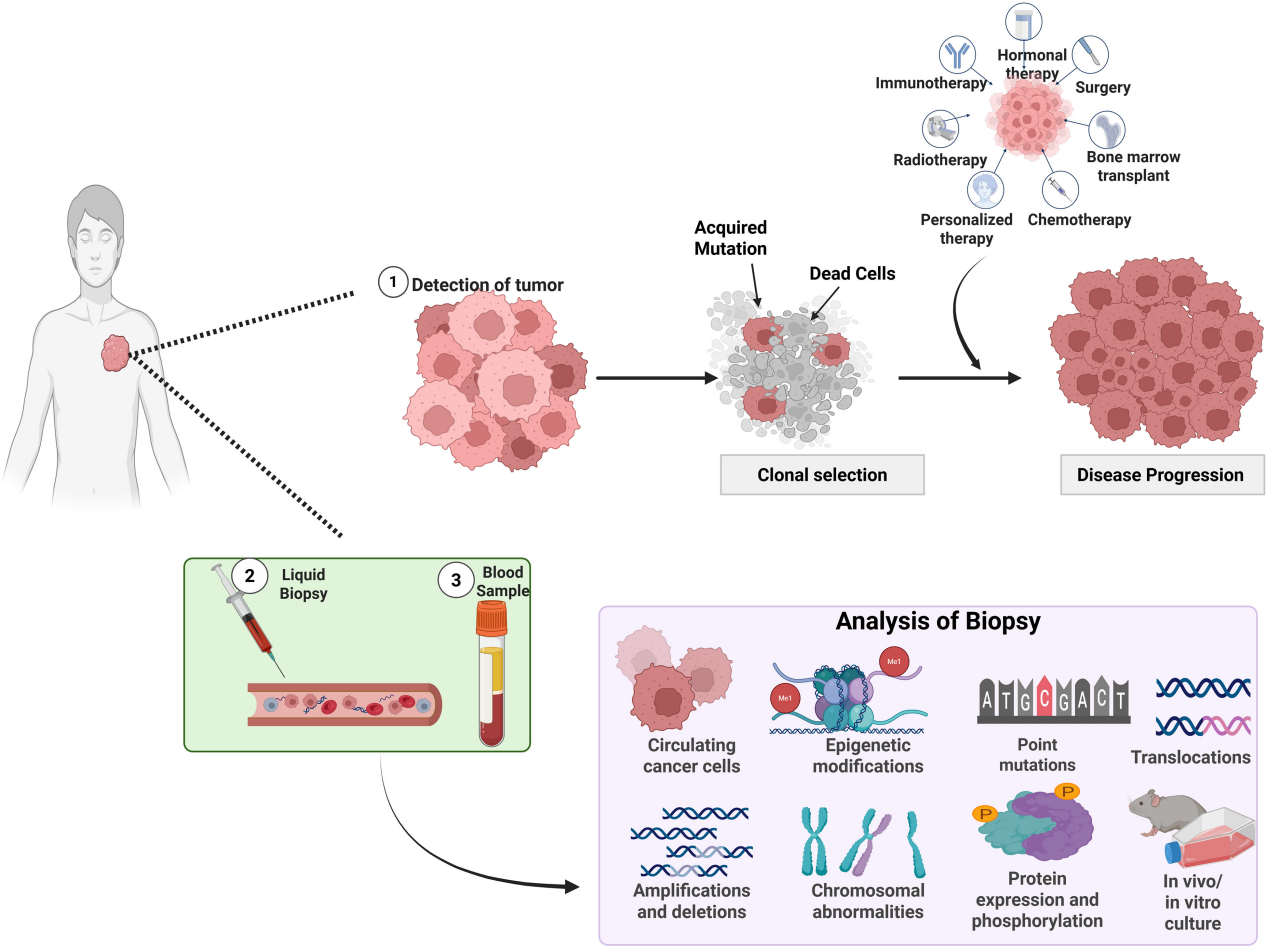
Clinical detection and monitoring of tumor heterogeneity and therapy-driven clonal evolution using tissue and liquid biopsy approaches. Schematic overview of tumor detection, therapeutic intervention, and heterogeneity-driven disease progression. Following initial tumor detection, therapeutic pressure promotes clonal selection by eliminating sensitive cells and favoring the survival of resistant subpopulations. Longitudinal monitoring using liquid biopsy and blood-based sampling enables the analysis of circulating tumor components and molecular alterations, including genetic mutations, epigenetic modifications, chromosomal abnormalities, and protein-level changes, providing insights into tumor evolution and resistance mechanisms. ctDNA, circulating tumor DNA.

However, ctDNA is also shaped by heterogeneity-related biases, including variable DNA shedding across lesions and underrepresentation of tumors in anatomically protected sites such as the central nervous system (160). Consequently, ctDNA and tissue-based profiling capture distinct but complementary dimensions of tumor heterogeneity. These concepts are schematically illustrated in **Figure 2** highlighting how therapy-driven clonal selection can be longitudinally tracked using tissue and liquid biopsy approaches.

#### 4.1.3 Prognostic implications of heterogeneity

Quantitative measures of intratumoral heterogeneity including sub clonal diversity, phylogenetic branching, and copy number variability are consistently associated with adverse clinical outcomes across cancer types (26). High heterogeneity reflects increased evolutionary potential, enabling rapid adaptation under therapeutic pressure. Similarly, tumors enriched for cancer stem like populations or exhibiting pronounced phenotypic plasticity demonstrate higher relapse rates and inferior survival in breast cancer, glioblastoma, and colorectal cancer (161–164). These prognostic associations establish heterogeneity not only as a mechanistic driver of resistance but also as a clinically meaningful biomarker of disease aggressiveness.

### 4.2 Impact of tumor heterogeneity across treatment modalities

The diagnostic and prognostic challenges described above translate directly into therapeutic vulnerability across chemotherapy, targeted therapy, and immunotherapy, where heterogeneity undermines uniform and durable responses. Chemotherapy resistance reflects both cellular and microenvironmental diversity within tumors (34). Clinically, Spatial heterogeneity contributes to partial responses and residual disease that frequently seeds relapse, helping explain why combination chemotherapy improves response rates yet rarely achieves durable remission in most solid tumors (133, 165). Targeted therapies face analogous constraints. Although initial responses can be profound, therapeutic benefit is often limited by the emergence of heterogeneous resistance patterns across tumor cell populations (5, 166). Together, these modality-specific examples illustrate how heterogeneity acts as a common constraint on therapeutic durability, necessitating treatment paradigms that anticipate evolutionary escape rather than react to it after progression.

## 5 Strategies to overcome heterogeneity-driven resistance

Tumor heterogeneity spans multiple biological dimensions, translational advances have converged on a subset of heterogeneity features that are currently most actionable in the clinic. These include adaptive signaling plasticity, pre-existing genetic diversity and microenvironmental protection mediated by stromal and myeloid compartments, and reversible epigenetic drug-tolerant states. Therapeutic strategies discussed in this section are therefore organized around these high-priority resistance axes, emphasizing interventions with demonstrated or emerging clinical feasibility rather than theoretical vulnerability alone. Or Accordingly, this section focuses on therapeutic strategies aligned with the most clinically actionable heterogeneity features outlined above, rather than attempting to target all theoretical sources of resistance.

**Figure 3:**
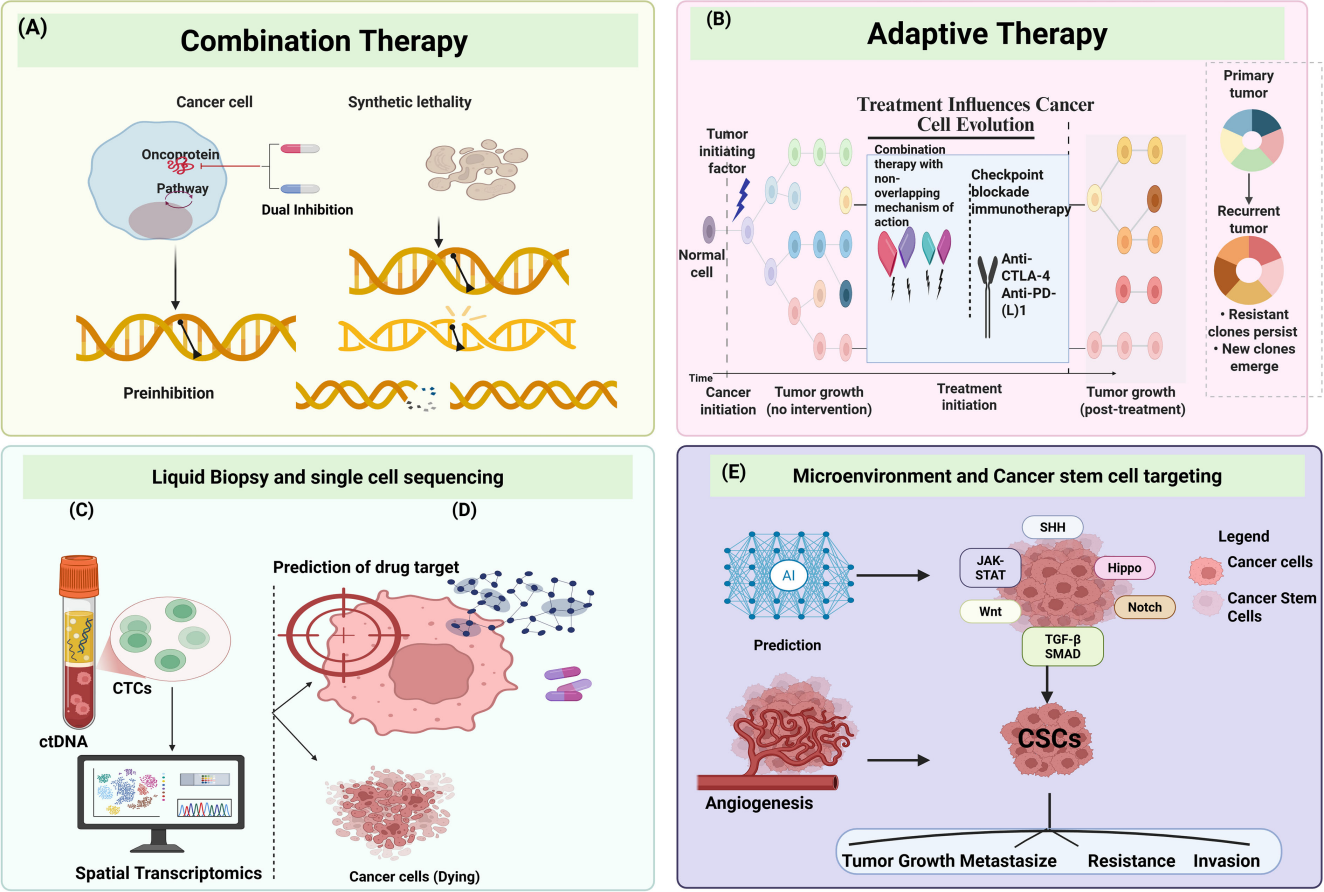
Therapeutic strategies to overcome tumor heterogeneity–driven resistance. **(A)** Combination therapy approaches exploit pathway co-dependencies and synthetic lethality to suppress heterogeneous cancer cell populations. **(B)** Adaptive therapy modulates treatment intensity over time to constrain clonal evolution and delay the expansion of resistant clones. **(C, D)** Liquid biopsy, single-cell sequencing, spatial transcriptomics, and artificial intelligence-based analyses enable real-time monitoring of tumor evolution and prediction of drug targets, identification of resistant subpopulations, and informed therapeutic adaptation, and **(E)** Microenvironment and cancer stem cell targeting. Tumor–stromal interactions, angiogenesis, and cancer stem cell (CSC) signaling pathways (e.g., WNT, NOTCH, TGF-β, JAK–STAT) sustain phenotypic plasticity and resistance. Integrative strategies combining microenvironmental modulation, CSC targeting, and AI-assisted prediction aim to suppress tumor growth, invasion, metastasis, and therapeutic failure. CSCs, cancer stem cells; ctDNA, circulating tumor DNA; CTCs, circulating tumor cells.

As detailed in Sections 3 and 4, resistance reflects the coordinated contribution of tumor-intrinsic and microenvironmental processes rather than a single dominant mechanism. Accordingly, the heterogeneity classes described here provide a biological rationale for combination, adaptive, and tumor microenvironment–targeted treatment strategies (**Figure 3**). An integrated overview of emerging therapeutic and clinical approaches designed to address tumor heterogeneity is shown in **Figure 3**.

### 5.1 Rational combination therapies to suppress pre-existing heterogeneity

Rational combination therapy represents a primary strategy to suppress resistant subclones present before treatment initiation by simultaneously targeting multiple tumor vulnerabilities. Vertical pathway inhibition has demonstrated clear clinical benefit, exemplified by combined RAF–MEK inhibition in BRAF-mutant melanoma, which suppresses MAPK feedback signaling, delays clonal escape, and significantly improves overall survival compared with BRAF inhibitor monotherapy (167, 168). Complementary horizontal pathway inhibition constrains compensatory signaling routes activated under therapeutic pressure, as shown by PI3K–CDK4/6 inhibition in hormone receptor positive breast cancer (169–171) and androgen receptor PARP inhibitor combinations in metastatic prostate cancer exploiting synthetic lethality (172, 173). Recent advances emphasize upfront co-targeting of oncogenic drivers and tissue-specific adaptive feedback circuits. Notably, KRAS-G12C inhibitors combined with EGFR antibodies in colorectal cancer directly counter rapid EGFR-mediated feedback activation that limits monotherapy efficacy (174). Representative clinical studies of rational combination strategies across solid tumors are summarized in **Table 2**, highlighting improvements in progression-free and event-free survival, often accompanied by increased toxicity. The mechanistic basis of these approaches is illustrated (**Figure 3A**). While effective at suppressing dominant resistance pathways early, combination regimens alone do not fully address the dynamic evolution of resistance during prolonged treatment.

**Table 2:** Summarizes representative clinical studies evaluating rational combination therapies designed to suppress heterogeneity-driven resistance mechanisms across solid tumors.

Combination Strategy	Cancer Type	Key Trial(s) / Study	Outcomes & Insights	Ref.
EGFR-TKI + Chemotherapy	Advanced EGFR-mutant NSCLC	Network meta-analysis (osimertinib + chemotherapy, lazertinib + amivantamab)	↑ PFS vs EGFR-TKI alone: higher adverse events.	(63, 73)
PARP inhibitor + Chemotherapy	BRCA1/2 germline breast cancer (neoadjuvant)	PARTNER trial (olaparib + carboplatin/paclitaxel)	No pCR difference; at 36 months ↑ , EFS (96.4% vs 80.1%), OS, BC-specific survival.	(175)
PARP inhibitor Maintenance	Advanced ovarian cancer (HRD / BRCA)	Meta-analysis of RCTs	↑ PFS, esp. in BRCA/HRD; OS benefit unclear; ↑ toxicity (grade ≥3 AEs)	(176)
PARP inhibitors + Chemotherapy vs Chemotherapy Alone	Triple-negative breast cancer (TNBC)	Systematic review/meta-analysis of RCTs	↑ PFS (HR ~0.83); moderate OS trend (NS); ↑ adverse events → risk-benefit balance	(176)
Immunotherapy + Chemotherapy Post-TKI Progression	NSCLC after osimertinib progression	Retrospective single-center study	↑ benefit with anti-PD-1/PD-L1 + chemo vs chemo alone; safety acceptable (mostly grade 1–2 AEs).	(177)
ICIs + multi-targeted TKIs	Advanced NSCLC (second- or later-line)	Recent meta-analysis	ORR ~26.4%; DCR ~80.7%; shorter mPFS (~3.17 mo) in EGFR/ALK/ROS1-mutants; common AEs: HTN.	(178)

EGFR-TKI, epidermal growth factor receptor tyrosine kinase inhibitor; NSCLC, non-small cell lung cancer; PARP, poly(ADP-ribose) polymerase; BRCA1/2, breast cancer susceptibility genes 1 and 2; HRD, homologous recombination deficiency; TNBC, triple-negative breast cancer; ICI, immune checkpoint inhibitor; TKI, tyrosine kinase inhibitor; PD-1, programmed cell death protein 1; PD-L1, programmed death-ligand 1; PFS, progression-free survival; OS, overall survival; EFS, event-free survival; ORR, objective response rate; DCR, disease control rate; mPFS, median progression-free survival; AEs, adverse events; HTN, hypertension.

↑ indicates increased response or improvement in the indicated clinical outcome.

Sequential therapy applies predefined treatment sequences to extend disease control following the emergence of resistance (179, 180). This approach is well established in EGFR-mutant non–small cell lung cancer, where first or second-generation EGFR inhibitors are followed by third-generation Osimertinib upon emergence of T790M mediated resistance, extending disease control despite eventual limitations imposed by spatial heterogeneity (181–183). Adaptive therapy modulates treatment intensity over time based on disease burden and treatment response. In metastatic castration-resistant prostate cancer, PSA-guided adaptive abiraterone dosing reduced cumulative drug exposure and significantly prolonged time to progression compared with continuous therapy (184, 185). Similar strategies using intermittent BRAF/MEK inhibition in melanoma have delayed resistance in preclinical and early clinical studies (186). These adaptive strategies have demonstrated in (**Figure 3B**) the feasibility of non-continuous dosing schedules in selected clinical contexts. Both sequential and adaptive strategies rely on longitudinal molecular monitoring, including serial tissue sampling and circulating tumor DNA analysis (**Figure 3C**), to guide treatment modulation (187). Together, these approaches highlight the clinical potential of treatment scheduling strategies informed by longitudinal disease monitoring.

### 5.2 Sequential and adaptive therapy: modulating evolutionary pressure

While the combination regimens summarized in **Table 2** improve disease control, their toxicity profiles highlight the limitations of sustained maximal inhibition and the need for evolution-informed treatment strategies. Although effective at suppressing baseline heterogeneity, combination therapies do not fully address dynamic tumor adaptation under prolonged therapeutic pressure. From a translational standpoint, evolutionary and treatment-induced heterogeneity represents one of the most actionable dynamic resistance axes, as it can be therapeutically modulated through treatment scheduling, dosing intensity, and real-time molecular monitoring rather than additional drug classes alone.

**Figure 4:**
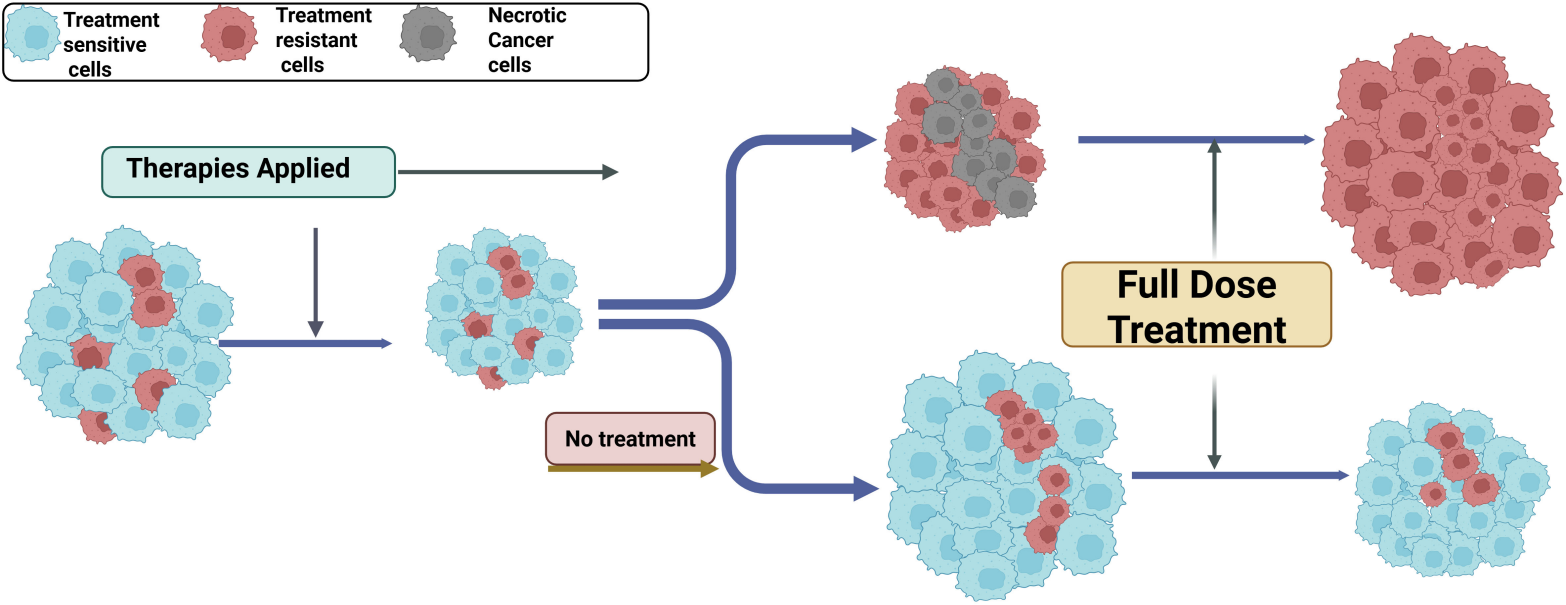
Therapy-induced clonal selection and evolutionary bottlenecks. Conceptual schematic illustrating how therapeutic pressure reshapes intratumoral heterogeneity through Darwinian selection. A heterogeneous tumor initially contains treatment-sensitive cells (blue), pre-existing resistant subclones (red), and necrotic regions (gray). Upon therapy application, sensitive cells are preferentially eliminated, while resistant subclones survive and expand. Continuous full-dose treatment accelerates clonal sweeps, leading to dominance of resistant populations and eventual treatment failure. In contrast, treatment modulation or drug holidays preserve a population of sensitive cells that competitively suppress resistant clones, thereby delaying resistance emergence. This framework provides the evolutionary basis for adaptive and evolution-informed therapy strategies.

Sequential and adaptive therapy models explicitly incorporate evolutionary principles by modulating selective pressure over time rather than applying continuous high-intensity treatment (188). These approaches are enabled by longitudinal molecular profiling, including serial tissue sampling and circulating tumor DNA analysis, which capture clonal dynamics in real time (**Figure 3C****).** Sequential therapy is well established in oncogene-driven cancers, particularly EGFR-mutant non–small cell lung cancer, where first or second-generation EGFR tyrosine kinase inhibitors are followed by Osimertinib upon emergence of T790M-mediated resistance (181–183). This strategy exploits predictable evolutionary trajectories but is ultimately constrained by spatial and inter-lesional heterogeneity. Representative clinical examples of sequential and adaptive therapeutic strategies designed to counteract heterogeneity-driven evolutionary resistance are summarized in **Table 3**. Adaptive therapy further extends this framework by preserving drug-sensitive cells to competitively suppress resistant clones (192). In metastatic castration-resistant prostate cancer, PSA-guided adaptive abiraterone dosing significantly reduced cumulative drug exposure while extending time to progression compared with continuous therapy (184, 185). Similar principles are being explored in BRAF-mutant melanoma using intermittent BRAF/MEK inhibition (193, 194). These adaptive paradigms, illustrated in **Figure 4B**, aim to regulate selective pressure rather than eradicate all tumor cells, reframing therapy as a regulator of tumor ecology.

**Table 3:** Representative clinical examples of sequential and adaptive therapy strategies designed to counteract heterogeneity-driven evolutionary resistance.

Strategy	Cancer Type	Clinical Example	Outcome	Ref.
Sequential Therapy	EGFR-mutant NSCLC	Sequential use of 1st/2nd-gen TKIs (erlotinib, gefitinib) → 3rd-gen osimertinib	Prolonged PFS and OS by delaying the emergence of resistant EGFR clones.	(189)
Adaptive Therapy	Prostate Cancer (mCRPC)	Adaptive abiraterone dosing guided by PSA dynamics	Reduced drug use by ~47% and extended time to progression vs. continuous therapy.	(190)
Adaptive Therapy	Melanoma (BRAF-mutant)	Intermittent dosing of BRAF/MEK inhibitors	Delayed resistance and improved response durability in preclinical and early-phase trials.	(191)
Sequential/Adaptive Monitoring	NSCLC, Prostate Cancer	Real-time ctDNA in NSCLC (EGFR T790M, KRAS) or PSA tracking in mCRPC	Enabled early detection of resistant clones and timely treatment switching/adaptation.	(189)
Adaptive Therapy	Prostate Cancer (mCRPC)	Evolution-based adaptive dosing of abiraterone	Prolonged response duration and identified strategies to further improve outcomes.	(190)

NSCLC, non-small cell lung cancer; EGFR, epidermal growth factor receptor; TKI, tyrosine kinase inhibitor; mCRPC, metastatic castration-resistant prostate cancer; PSA, prostate-specific antigen; BRAF, B-Raf proto-oncogene serine/threonine kinase; MEK, mitogen-activated protein kinase kinase; ctDNA, circulating tumor DNA; KRAS, Kirsten rat sarcoma viral oncogene homolog; PFS, progression-free survival; OS, overall survival.

### 5.3 Targeting the TME to disrupt extrinsic resistance

Tumor-intrinsic targeting alone is insufficient when resistance is reinforced by stromal and immune compartments Microenvironmental targeting strategies aim to dismantle extrinsic protective niches that buffer cancer cells from therapy (195). Vascular normalization using anti-VEGF agents enhances drug delivery and immune-cell infiltration, thereby improving systemic therapy efficacy (196). This principle is clinically validated by atezolizumab–bevacizumab in hepatocellular carcinoma and pembrolizumab-axitinib in renal cell carcinoma, where vascular modulation synergizes with immunotherapy to improve survival (197). Parallel approaches seek to reprogram cancer-associated fibroblast signaling, particularly TGF-β–driven stromal exclusion, and to suppress immunosuppressive myeloid niches via CSF1R inhibition (198). Emerging cellular therapies further integrate microenvironmental modulation. Armored CAR-T cells and bispecific T-cell engagers are now being combined with vascular or stromal-targeting agents, with early 2024–2025 trials reporting encouraging activity in selected solid tumors previously considered refractory to cellular immunotherapy (199, 200). Myeloid-mediated immunosuppression and stromal exclusion emerge as key actionable microenvironmental resistance axes, given that are particularly amenable to therapeutic intervention, supported by the clinical availability of VEGF-targeting agents, CSF1R inhibitors, and immunomodulatory combination strategies. **Figure 3D** provides an integrated overview of microenvironmental and cancer stem cell targeting strategies that disrupt extrinsic resistance mechanisms outlined in Section 3.4. Microenvironmental and CSC-targeting strategies) directly address the extrinsic resistance mechanisms outlined in Section 3.4. and targeting microenvironmental cues and cancer stem cell programs that sustain resistance are summarized in **Figure 3D**). Beyond genetic resistance, cellular plasticity and stem-like states represent critical non-genetic reservoirs of persistence that must be addressed to prevent relapse (123, 201).

### 5.4 Targeting cellular plasticity and non-genetic resistance states

Although phenotypic plasticity is inherently dynamic, epigenetic regulators that stabilize drug-tolerant states are increasingly druggable, positioning epigenetic therapy as one of the most promising near-term strategies to suppress non-genetic heterogeneity.

From a translational standpoint, epigenetic plasticity represents the most immediately actionable component of cellular state heterogeneity, as chromatin-modifying enzymes are directly targetable with clinically available inhibitors. Genetic targeting alone cannot eradicate phenotypically plastic tumor states that drive relapses. Strategies aimed at cancer stemness, and epigenetic reprogramming seek to eliminate the reservoirs of adaptive resistance that persist during therapy (116, 202). Cancer stem cell–directed approaches include Hedgehog pathway inhibition, WNT/NOTCH targeting, metabolic disruption of oxidative phosphorylation, and antigen-specific strategies such as CD133-directed CAR-T cells (203, 204). These interventions aim to prevent repopulation of the tumor by therapy-tolerant clones following initial response. Epigenetic therapies provide a complementary strategy by reversing drug-tolerant persister states. DNMT, HDAC, EZH2, and BET inhibitors disrupt chromatin programs that sustain reversible resistance, resensitizing tumors to targeted agents and immunotherapy (109). Combination trials incorporating epigenetic modulators are rapidly expanding across solid tumors, reflecting growing recognition that non-genetic resistance is a critical bottleneck to durable response (205).

### 5.5 AI–guided prediction and evolution-aware trial design

Artificial intelligence and computational modeling increasingly serve as the analytical backbone for heterogeneity-aware oncology by integrating multi-omics, imaging, and longitudinal clinical data into predictive frameworks (1) Machine-learning models that combine ctDNA kinetics, radiomic features, and clinical variables now forecast resistance emergence and treatment failure in non–small cell lung cancer and breast cancer, enabling pre-emptive therapeutic intervention rather than reactive salvage strategies (206, 207). These approaches directly operate the evolutionary dynamics described in earlier sections by anticipating clonal shifts before they manifest clinically. Heterogeneity-aware clinical trial designs translate these computational advances into practice. Conventional trials assume relatively homogeneous tumor behavior and static treatment response assumptions that conflict with the spatial and temporal complexity of cancer evolution. In contrast, modern trial frameworks incorporate molecular stratification, longitudinal monitoring, and adaptive decision-making to align treatment with evolving tumor biology (208, 209). Umbrella trials stratify patients with a single cancer type into parallel arms based on molecular alterations, enabling efficient evaluation of targeted therapies within heterogeneous diseases (210). Landmark examples include LUNG-MAP in squamous NSCLC and I-SPY 2 in breast cancer, which integrates adaptive randomization to rapidly identify effective biomarker therapy matches (211). Basket trials extend this logic across histologies, as demonstrated by durable responses to TRK inhibitors in NTRK fusion–positive cancers and by recent KRAS-G12C inhibitor trials that reveal tissue-specific adaptive resistance mechanisms (212, 213). Adaptive and platform trials further enhance flexibility by allowing therapies to enter or exit based on interim efficacy or resistance signals. When paired with liquid biopsy monitoring, these designs enable real-time therapeutic redirection, exemplified by ctDNA guided switching to osimertinib upon emergence of EGFR T790M mutations in NSCLC (214) Platform trials such as NCI-MATCH and GBM AGILE maintain perpetual infrastructures optimized for testing combination and sequencing strategies in highly heterogeneous tumors (215, 216). Window-of-opportunity and evolution-informed trials provide additional resolution by capturing early pharmacodynamic effects, clonal selection, and immune remodeling within intact tumor ecosystems (185, 217, 218). These designs have yielded critical insights into adaptive responses to endocrine therapy, immunotherapy, and androgen deprivation, informing treatment schedules explicitly designed to delay resistance (219, 220). Together, these trial paradigms transform clinical research from static hypothesis testing into dynamic learning systems that integrate tumor heterogeneity, evolutionary principles, and real-time molecular feedback forming a necessary bridge between precision oncology and evolutionary cancer medicine. Taken together, these strategies underscore a paradigm shift from static precision oncology toward evolution-informed cancer medicine, in which treatment is continuously adapted to tumor heterogeneity and dynamic resistance trajectories. To translate these diagnostic limitations into clinical context, several prospective and retrospective studies have explicitly interrogated intratumoral and intertumoral heterogeneity using multi-region tissue sampling, circulating tumor DNA, and advanced imaging modalities across cancer types, (**Table 4**). Collectively, these trials demonstrate the feasibility of integrating spatial sampling, molecular profiling, and longitudinal monitoring into clinical study design, providing a foundation for evolution informed therapeutic strategies (227). Together with adaptive treatment paradigms and combination strategies, heterogeneity-focused clinical trials represent a critical step toward operationalizing evolutionary oncology in routine cancer care. Despite substantial progress, therapeutic strategies aimed at overcoming heterogeneity-driven resistance remain constrained by several important limitations. Many combination and microenvironment-targeting approaches are associated with increased toxicity, narrow therapeutic windows, and context-dependent efficacy that varies across tumor types and disease stages. Moreover, robust biomarkers capable of prospectively identifying which heterogeneity axis dominates resistance in individual patients are still lacking. As a result, most current strategies are applied empirically rather than dynamically tailored to evolving tumor states. These challenges underscore the need for improved patient stratification, longitudinal monitoring, and adaptive treatment frameworks to translate heterogeneity-aware strategies into durable clinical benefit.

**Table 4:** Representative clinical studies evaluating tumor heterogeneity using multi-region tissue sampling, liquid biopsy, and multimodal imaging approaches.

Cancer type	Study	Status	Study design	Clinical Findings	Ref.
NSCLC; Afatinib	Deciphering Afatinib Response and Resistance with Intratumor Heterogeneity	Completed	Interventional	Demonstrated that intratumoral heterogeneity contributes to differential responses to EGFR inhibitors and the emergence of resistant clones.	(190)
NSCLC; MPDL3280A, Vemurafenib, Alectinib, Trastuzumab	Deciphering Antitumor Response and Resistance with Intratumor Heterogeneity	Active, not recruiting	Interventional	Identified heterogeneous resistance mechanisms and variable therapeutic responses among tumor subclones.	(221)
Melanoma	Exome Sequencing and ctDNA to Assess Tumor Heterogeneity in BRAF-Mutant Melanoma	Completed	Observational	Revealed spatial and temporal heterogeneity and enabled tracking of resistance mutations during therapy.	(222)
Prostate Cancer; Targeted biopsies	Prostate Cancer Genomic Heterogeneity	Completed	Interventional	Demonstrated significant genomic divergence among tumor regions influencing treatment outcomes.	(223)
Glioblastoma	Patient-Derived Glioma Stem Cell Organoids	Active, not recruiting	Observational	Showed preservation of intratumoral heterogeneity and utility for modeling therapeutic responses.	(224)
Differentiated Thyroid Cancer	Impact of BRAFV600E Intratumor Heterogeneity in Thyroid Cancer	Unknown	Observational	Demonstrated that heterogeneous mutation distribution affects prognosis and therapeutic response.	(225)
Rectal Cancer; MRI-based diagnostics	Multimodal MRI for Prognostic Assessment of Rectal Cancer	Not yet recruiting	Observational	Imaging-based heterogeneity metrics correlate with treatment response and clinical outcomes.	(226)

NSCLC, non-small cell lung cancer; EGFR, epidermal growth factor receptor; ctDNA, circulating tumor DNA; MRI, magnetic resonance imaging; BRAFV600E, BRAF V600E mutation; MPDL3280A, programmed death-ligand 1 (PD-L1) inhibitor.

## 6 Conclusions: toward evolution informed precision oncology

**Figure 5:**
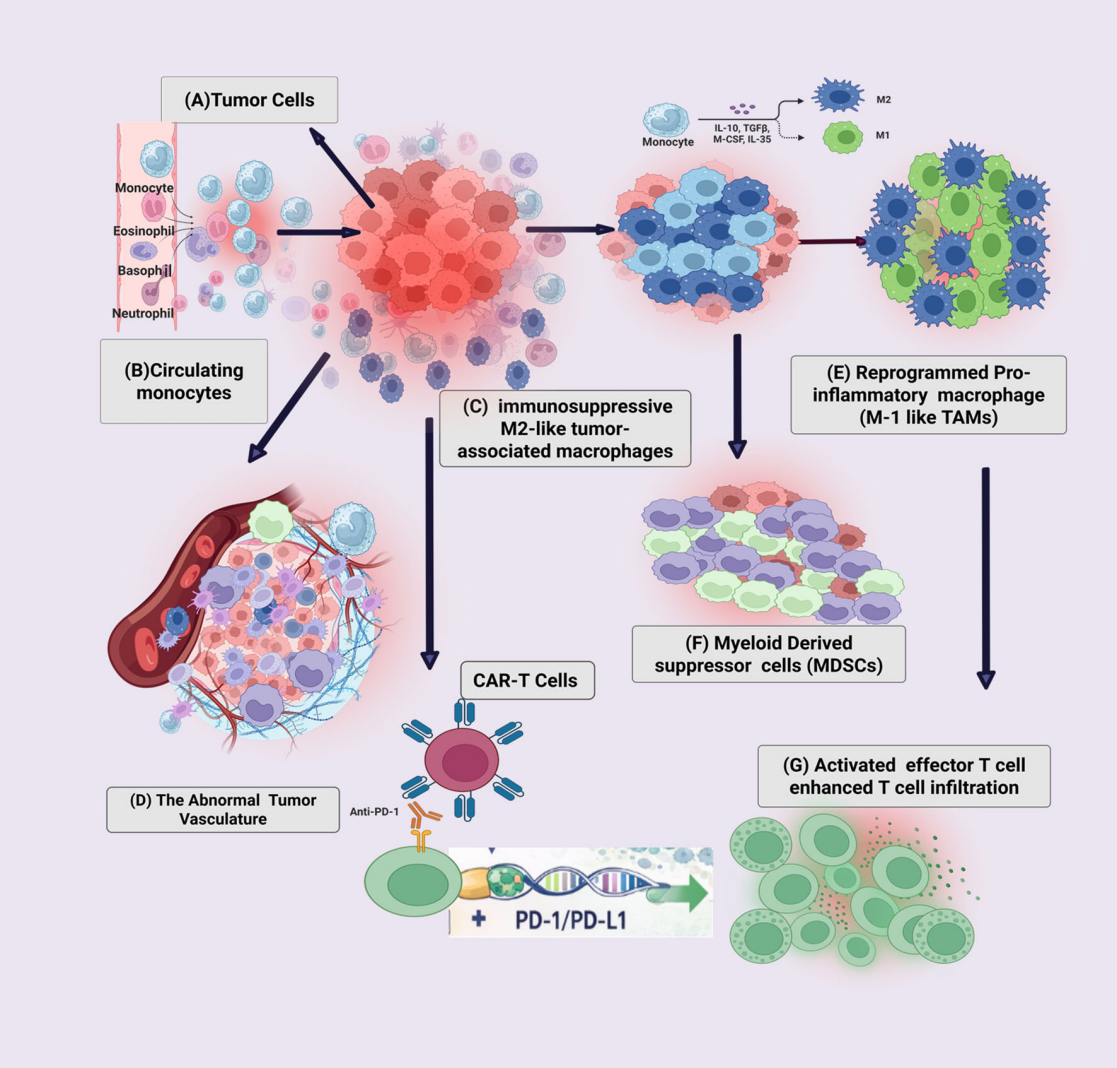
Myeloid reprogramming strategies to overcome tumor-associated immune suppression. Schematic illustrating the dynamic remodeling of the tumor microenvironment by myeloid cells and therapeutic intervention. **(A)** Tumor cells establish an immunosuppressive niche through the release of cytokines and growth factors. **(B)** Circulating monocytes are recruited from the bloodstream into the tumor microenvironment. **(C)** Within tumors, monocytes differentiate into immunosuppressive M2-like tumor-associated macrophages (TAMs) that inhibit antitumor immunity and promote tumor progression. **(D)** Abnormal, VEGF-driven tumor vasculature facilitates myeloid cell recruitment while limiting effective T-cell infiltration. **(E)** Therapeutic reprogramming converts suppressive M2-like TAMs into pro-inflammatory M1-like macrophages with enhanced antigen presentation and immune-stimulatory functions. **(F)** Myeloid-derived suppressor cells (MDSCs) further dampen adaptive immune responses through metabolic and checkpoint-mediated mechanisms. **(G)** Combined myeloid reprogramming with CAR-T cell therapy and PD-1/PD-L1 blockade restores T-cell effector function, leading to increased T-cell infiltration and improved antitumor activity.

Tumor heterogeneity has profound implications for therapeutic response, resistance, and disease progression across cancer types. The cumulative evidence reviewed here demonstrates that therapeutic failure is rarely attributable to inadequate target inhibition alone; instead, it reflects the combined impact of pre-existing diversity, reversible phenotypic plasticity, and extrinsic microenvironmental protection operating within individual tumors (228). These features generate complex resistance landscapes that undermine uniform and durable responses to both cytotoxic and targeted therapies. Importantly, resistance emerges through multiple, coexisting processes that operate on overlapping spatial and temporal scales within the same patient (229). Pre-existing subclones, therapy-induced clonal evolution, adaptive transcriptional reprogramming, and microenvironment-mediated buffering frequently coexist generating divergent resistance mechanisms across tumor sites and over time (230). This complexity challenges static treatment paradigms and limits the effectiveness of sequential monotherapy approaches in heterogeneous disease settings., The tumor microenvironment is not merely a passive scaffold for resistance, but an active and dynamic contributor that shapes both tumor evolution and treatment response and disease persistence. Through Immune and stromal remodeling, as well as metabolic constraint, it impose selective pressures that influence therapeutic sensitivity, particularly in the context of immunotherapy. In this context, myeloid populations, including neutrophils, macrophages, monocytes, and dendritic cells, emerge as key regulators of immune escape and therapeutic persistence by reinforcing immune-suppressive niches, limiting effective antigen presentation, and promoting tissue remodeling that supports resistant cellular states (shown in **Figure 5**). Consequently, durable cancer control cannot be achieved through isolated targeting of dominant clones alone. Instead therapeutics strategies that must anticipate and constrain resistance trajectories, while also accounting for microenvironmental and immune selection pressures. Future clinical trials should incorporate evolutionary modeling concepts (**Figure 2**) to enable adaptive treatment strategies capable of responding to tumor dynamics over time.

## 7 Future perspectives

Precision oncology must shift from a purely mutation-centric framework toward an evolution-aware therapeutic model. Rational upfront combination therapies, adaptive dosing schedules, and sequential strategies informed by real time molecular monitoring represent critical steps in this transition. Liquid biopsy technologies, particularly ctDNA analysis, provide a practical means to track clonal dynamics, detect emergent resistance, and guide timely therapeutic intervention (231). When integrated with spatial profiling, single-cell analyses, and computational modeling, these tools enable a systems-level view of tumor evolution that aligns therapeutic decision-making with biological reality (232). Equally important is the recognition that non-genetic mechanisms epigenetic plasticity, lineage switching, metabolic adaptation, and microenvironmental crosstalk are not secondary resistance pathways but fundamental drivers of persistence and relapse (233). Therapeutic strategies targeting CSC-like states (234), drug-tolerant persisters (235), stromal signaling axes, and immune-suppressive niches are essential complements to genomic targeting (236, 237). A major next step will be to more precisely resolve how myeloid heterogeneity and functional plasticity contribute to spatially localized resistance programs, particularly in tumors arising in barrier-associated tissues where immune tolerance mechanisms and inflammatory cues can strongly shape therapy response (238). High-resolution profiling of granulocyte and mononuclear phagocyte subsets, combined with longitudinal sampling, will help identify actionable immune–metabolic circuits that sustain resistant tumor states. Ultimately, the convergence of tumor-intrinsic and microenvironmental interventions, including strategies aimed at remodeling or reprogramming myeloid-driven suppressive niches, offers an opportunity to collapse evolutionary escape routes rather than simply delay them.

Importantly, while evolution-informed and heterogeneity-aware therapeutic strategies offer a compelling framework for improving cancer outcomes, they do not eliminate resistance but instead aim to delay, redirect, or constrain evolutionary escape (239). Tumor heterogeneity is unlikely to be fully eradicated; rather, its clinical impact can be mitigated through rational combinations, adaptive scheduling, and integration of tumor-intrinsic and microenvironmental targeting. Realizing this potential will require closer alignment between biological insight, biomarker development, and trial design capable of capturing spatial and temporal tumor dynamics (240).

Thus, the central challenge moving forward is not simply identifying resistance mechanisms, but operationalizing heterogeneity into actionable, patient-specific therapeutic decisions.
